# PanCD44 Immunohistochemical Evaluation in Prostatectomies from Patients with Adenocarcinoma

**DOI:** 10.1155/2018/2061268

**Published:** 2018-02-26

**Authors:** Carlos Gustavo Hirth, Adriele Machado dos Santos, João Batista Gadelha de Cerqueira, Francisco Vagnaldo Fechine Jamacaru, Maria do Perpétuo Socorro Saldanha da Cunha, Conceição Aparecida Dornelas

**Affiliations:** ^1^Pathology Department, Federal University of Ceará, Fortaleza, CE, Brazil; ^2^Federal University of Ceará, Fortaleza, CE, Brazil; ^3^Urology Department, Federal University of Ceará, Fortaleza, CE, Brazil; ^4^Haroldo Juaçaba Hospital, Fortaleza, CE, Brazil

## Abstract

**Introduction:**

CD44 has been proposed as a prognostic marker and a stem cell marker but studies in patients with prostate cancer have yielded inconsistent results.

**Patients and Methods:**

Patients submitted to radical prostatectomy between 2008 and 2013 at a university hospital were followed with biannual serum PSA tests to determine the biochemical recurrence (BR). Archived paraffin blocks with neoplastic and nonneoplastic tissue were evaluated immunohistochemically for a panCD44 and MYC.

**Results:**

Sixty-nine patients completed follow-up and were included. CD44 positivity was observed in inflammatory cells (42%), nonneoplastic epithelium (39.7%), and neoplastic tissue (12.3%). In nonneoplastic tissues staining was observed in basal and luminal cells with the morphology of terminally differentiated cells. In neoplastic tissues, CD44 negativity was correlated with higher Gleason scores (Rho = −0.204; *p* = 0.042) and higher preoperative serum PSA levels when evaluated continuously (*p* = 0.029). CD44 expression was not associated with tumor stage (*p* = 0.668), surgical margin status (*p* = 0.471), or BR (*p* = 0.346), nor was there any association between CD44 and MYC expression in neoplastic tissue (*p* = 1.0).

**Conclusion:**

In the bulk of cells, the minority of cancer stem cells would not be detected by immunohistochemistry using panCD44. As a prognostic marker, its expression was weakly correlated with Gleason score and preoperative PSA level, but not with surgical margin status, tumor stage, or BR.

## 1. Introduction

The cell-surface glycoprotein CD44 plays a central role in cell-cell and cell-matrix adhesion [1–5]. Along with integrins and cadherins, CD44 promotes the fixation and maintenance of tissue integrity and participates in microenvironmental signal transduction [3], modulating cell growth, survival, differentiation, and mobility [3]. CD44 gene exons can be combined by alternative splicing to form several isoforms [6–8]. CD44s (standard), the smallest form of CD44, is universally expressed in vertebrates [3] and has been detected in lymphocytes, macrophages, granulocytes, fibroblasts, and epithelial cells [2]. There are at least 15 variants of CD44 (CD44v) with the same N- and C-terminal sequences but differing with regard to the central portion [2, 3, 9]. Its expression in prostate cancer is associated with reduced tumor aggressiveness [3, 10–12].

On the other hand, it has been hypothesized that stem cells in the basal epithelium are capable of self-renewal and differentiation into terminal cells [13, 14]. Cancer-initiating cells constitute a specific transformed rare cell population with unchecked proliferation, which—unlike normal stem cells—may display genetic unstability and heterogeneity, favoring tumor formation and growth [9, 15–17]. Thus, approximately 0.1% of tumor cells would be stem cells [18]. Several markers have been used to identify and isolate stem cells [19], especially CD44 combined or not with other markers. CD44-positive cells can proliferate over long periods and give rise to other cell types with significant metastatic and tumorigenic potential [20, 21]. In addition, CD44 expression has been associated with the presence neoplastic cells in the blood stream [22]. However, some authors believe the scope of CD44 expression in neoplasia is too wide to make it a useful stem cell marker [23].

MYC is one of the proteins regulated by CD44 [21]. MYC-encoded proteins are transcription factors, the expression of which is correlated with cell proliferation, differentiation, and tumorigenesis [24, 25]. This raises the possibility that the preference of MYC expression for basal prostate epithelium is evidence of the presence of stem cells [26].

The purpose of the present study was to evaluate the immunohistochemical expression of panCD44 in neoplastic and nonneoplastic prostate epithelium, determining its association with tumor aggressiveness (preoperative PSA level, Gleason score, tumor stage, surgical margin status, and biochemical recurrence) and C-MYC expression.

## 2. Patients and Methods

### 2.1. Study Population, Ethical Considerations, and Prognostic Factors

The sample consisted of all patients with localized prostate cancer (clinical stages T1 and T2) [27] submitted to radical prostatectomy between January 2008 and December 2013 at a referral hospital (Hospital Universitário Walter Cantídio/UFC) in Northeastern Brazil (Fortaleza, Ceará). The patients were followed clinically with biannual serum PSA tests between January 2008 and June 2016. Patients missing follow-up visits were excluded from the sample. The study protocol was approved by the institutional ethics committee and all patients gave their informed written consent. Biochemical recurrence (BR) was defined as a sustained (≥2 measurements) PSA level of ≥0.2 ng/mL after reaching PSA nadir [28, 29]. The data were entered in a protocol designed for the study, according to the time elapsed between surgery and BR or censoring. In the absence of BR, the patients were censored on the day of the last PSA measurement. Information on preoperative serum PSA levels was retrieved from the medical records. The histology slides were reviewed by a pathologist blinded to the clinical data. Modified Gleason scores were assigned following 2016 WHO guidelines [30]. The pathological stage was determined according to the 2010 AJCC Cancer Staging Manual [27], considering the criteria for extraprostatic extension and seminal vesicle involvement in the 2011 guidelines of the International Society of Urological Pathology (ISUP) [31, 32]. Neoplastic cells in contact with the india ink used in the processing of the specimen were considered evidence of compromised surgical margins [33].

### 2.2. Immunohistochemistry for CD44 and C-MYC

The immunohistochemical evaluation used archived paraffin blocks. Fragments were selected which contained adenocarcinoma, benign gland tissue, and specimens with highly variable Gleason scores (the higher, the better). Three-micrometer sections were placed on silanized slides according to standard laboratory procedures [34], followed by incubation at 60°C for one hour. Diaphonization, rehydration, and heat-induced epitope retrieval were performed with a Dako PT Link™ pretreatment module and Tris/EDTA buffer at pH 9.0 (Dako). Automated reactions were performed with an Auto Stainer (Dako™). CD44 reactions were performed with the clone SFF-2 (Santa Cruz, sc-65405, 2015, dilution 1/100, incubation 30 min). The reactions were evaluated semiquantitatively under an optical microscope (Nikon Eclipse E200). The samples were considered positive when the membrane stained with a total score of >3, as described elsewhere [35]: the proportion of positive cells in relation to the total sample was classified as 0 (0–5%), 1 (6–25%), 2 (26–75%), or 3 (76–100%), while intensity was classified as 0 (absent), 1 (weak, positivity observed at 400x), 2 (intermediate, positivity observed at 100x), or 3 (strong, positivity observed at 40x). MYC reactions were performed with the clone Y69 (Abcam, ab32072, 2015, dilution 1/100, incubation 60 min). The results were plotted as positive or negative for nonneoplastic epithelium, blood vessels, and neoplastic tissue [36].

### 2.3. Statistical Analysis

The statistical analysis was made with the software IBM SPSS v.22. The normality of the data was verified with the Shapiro-Wilk test, while the Mann–Whitney test was used to verify potential associations between CD44 expression and serum PSA levels. The frequency analysis of the categorical variables was done with Fisher's exact test and odds ratios (95% confidence interval). The Spearman test was employed to verify correlations between CD44s expression and Gleason patterns. Kaplan-Meier curves were generated and compared with the log rank test. Univariate Cox regression was used to detect associations between BR and CD44s expression. The level of two-tailed statistical significance was set at 5% (*p* ≤ 0.05).

## 3. Results

### 3.1. Patients

During the study period, 74 patients were submitted to radical prostatectomy. The criteria for clinical follow-up were met by 69 subjects (93.2%), with a mean follow-up time of 41 months (range: 2–89), during which 20 (29%) were diagnosed with BR. In four paraffin blocks, the neoplasia was exhausted (no immunohistochemical representation). The same occurred with one sample of benign epithelium.

### 3.2. CD44 and MYC

**Figure 1:**
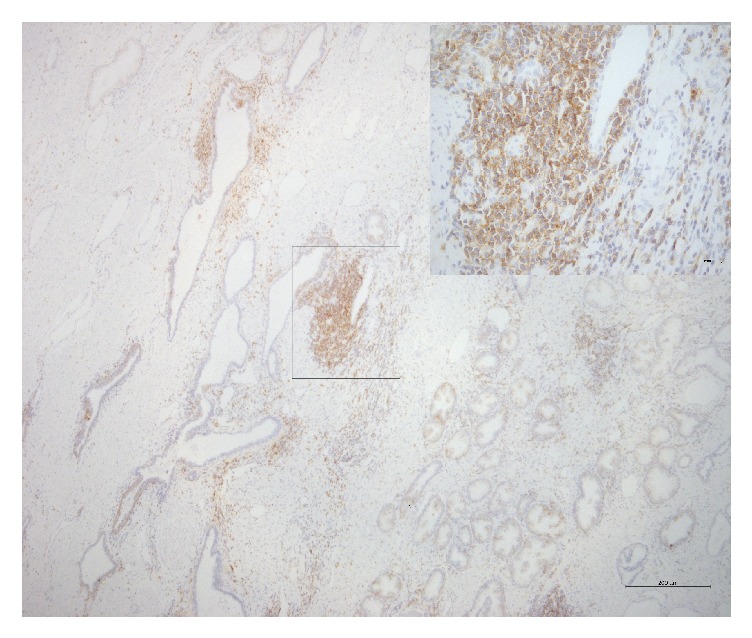
Photomicrograph of immunohistochemical staining for CD44 in stromal inflammatory cells. Staining was observed in the membrane and cytoplasm (magnification: 50x; detail: 400x).

**Figure 2:**
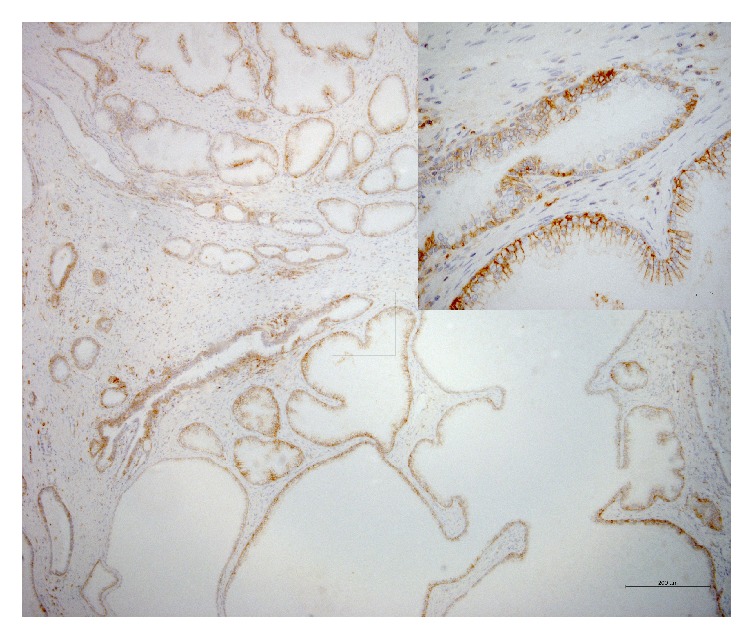
Photomicrograph of immunohistochemical staining for CD44 in nonneoplastic epithelium. Staining was basolateral in luminal cells and circumferential in basal cells. The intensity was grade 3 in over 75% of the cells (magnification: 100x; detail 400x).

**Figure 3:**
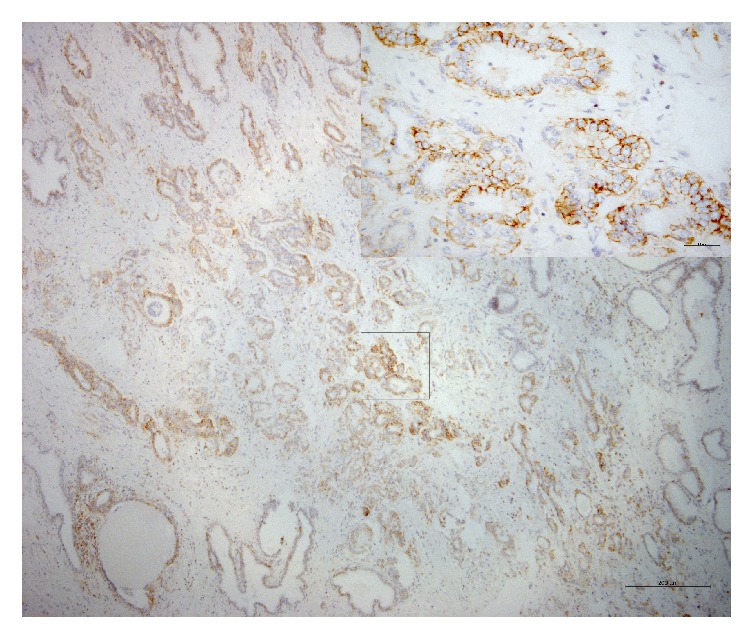
Photomicrograph of immunohistochemical staining for CD44 in neoplastic cells. Staining was basolateral. The intensity was grade 3 in over 75% of the cells (magnification: 50x; detail 400x).

The membrane and cytoplasm of inflammatory cells stained positive for CD44 (intensely and/or extensively) in 42% of the samples (Figure 1). As for normal epithelium (positivity: 39.7%), staining was circumferential in basal cells and basolateral in luminal cells (the latter displaying the morphology of terminally differentiated cells) (Figure 2). In neoplastic cells (positivity: 12.3%), staining was circumferential, basolateral, or focal (Figure 3).

The immunohistochemical findings for CD44 are summarized in Table 1. CD44 expression was statistically similar in inflammatory cells and benign epithelium (*p* = 0.862), but significantly weaker in neoplasia (*p* ≤ 0.001).

CD44 positivity in neoplastic cells was associated with lower preoperative PSA levels. Thus, the median level was 5.43 ng/mL (range: 4.16–16.35) in positive cases and 8.42 ng/mL (range: 3.06–94.3) in negative cases (*p* = 0.029). The CD44 staining score was inversely but weakly correlated with Gleason pattern (Rho = −0.204; *p* = 0.042), but the association was nonsignificant when the PSA values were categorized and the Gleason scores were stratified (Table 2).

**Figure 4:**
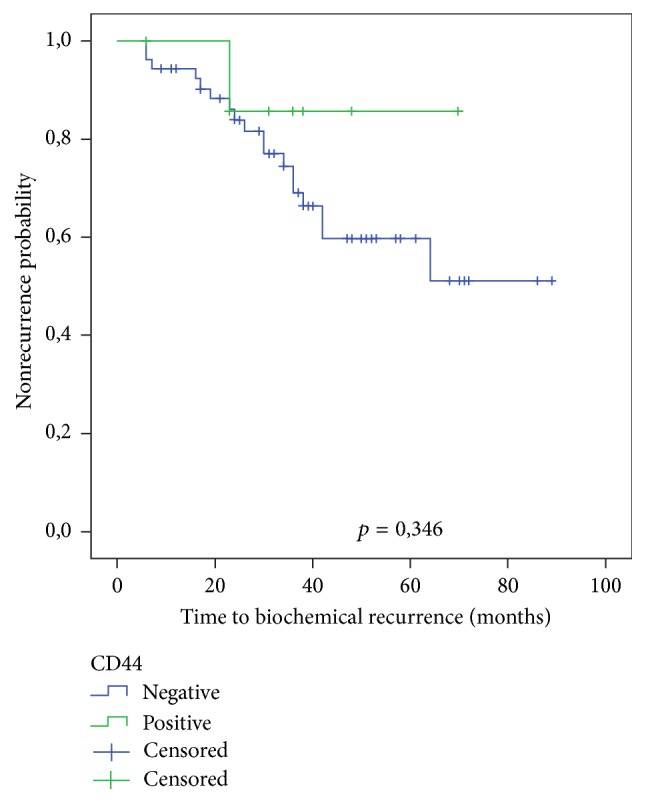
Kaplan-Meier curve showing the probability of biochemical recurrence according to CD44 positivity in neoplastic cells. The level of significance was evaluated with the log rank test.

The presence or absence of immunoexpression for CD44 was not associated with tumor stage, surgical margins, or BR (Table 2; Figure 4). The univariate Cox regression yielded a hazard ratio of 1.100 (95% IC: 0.446–2.710; *p* = 0.836) for the relation between CD44 expression and BR.

Nuclear staining for MYC was observed in rare endothelial cells (40.6% of cases), nonneoplastic basal epithelial cells (25%), and neoplastic cells (27.3%). MYC expression and CD44 expression were significantly associated in nonneoplastic epithelium, but not in neoplastic tissue.

## 4. Discussion

The ability of CD44 expression in biopsies and transurethral resections to predict tumor aggressiveness in prostatectomies had been evaluated [37, 38], as had the association of biopsies and transurethral resections with anatomopathological factors and tumor progression [39–41]. Some authors have focused on primary tumors and metastases [10, 42–45]. In this study, we focused on prostatectomies.

The detection of CD44 on immunohistochemistry may be approached in many different ways (Table S1). CD44 goes under a variety of names, data may be plotted in different forms, and different clones may be used. Over time, PSA detection techniques have become ultrasensitive, staging criteria have been revised, Gleason scores have been modified, and different cut-off values have been used to define BR. This makes studies highly heterogeneous and difficult to compare, compromising attempts at meta-analysis.

Cut-off values for CD44 generally indicate “superexpression” or “intense expression,” as in Aaltomaa et al. (2001) [41], in which positivity was defined as staining of 80% cells, below which associations were considered “weak.”

In our study, preoperative serum PSA levels were lower in CD44-positive patients than in CD44-negative patients, but the association was nonsignificant when PSA levels were stratified using 10 ng/mL as cut-off. An association between CD44 status and PSA level was also reported by Aaltoma et al. (2002) [46], but not by Kallakury et al. (1998) [47] and Tei et al. (2014) [35] who failed to detect an association when PSA levels were stratified (10 ng/mL).

CD44 positivity and Gleason score were correlated, showing a decline in CD44 expression from Gleason pattern 1 to Gleason pattern 5 [11], in our study with a weak Spearman correlation coefficient (−0.204) but a significant *p* value. However, when the Gleason scores were summed up and stratified, the association was nonsignificant. De Marzo et al. [10] tested a number of hypotheses using contingency tables to compare Gleason scores with CD44 expression in normal epithelium, intraepithelial neoplasia, and carcinoma and observed an inverse correlation between the patterns, especially Gleason 4 and 5. CD44 expression is no doubt weaker in neoplastic tissue than in healthy tissue and weaker in poorly differentiated neoplasia than in well-differentiated neoplasia, resembling the behavior of other adhesions molecules such as E-cadherin [48].

The lack of association between CD44 expression and tumor stage observed in this study matches the findings of several authors [22, 35, 46, 47], but not those of Lazari et al. (2013) [48] and Noordzij et al. (1997) [11]. The former focused on the loss of expression of cell adhesion molecules. The latter stratified CD44 levels and tumor stage into four groups and submitted the findings to the chi-squared test, but in one of the cells the expected value was under 5, compromising the statistical power of the test. In 2000, Vis and coworkers reviewed their findings from 1997 and republished the Spearman correlation coefficient −0.49 (*p* = 0.001) [49] as evidence of an association between CD44 and tumor stage.

Matching the results of other authors [35, 46], in our study no association between CD44 staining and surgical margin status was observed.

Finally, CD44 was not associated with BR. Only one of the reviewed studies found an association with BR in all analyses (Noordzij et al., 1997) [11]. In the remainder, no such association was found in the univariate analysis or, if found, was nonsignificant in the multivariate analysis [22, 29, 35, 46, 47] (Table S2). Noordzij et al. (1997) [11] and Vis et al. (2000) [49] also found CD44 expression to be related to clinical progression and specific mortality.

A large proportion of neoplastic and nonneoplastic cells express CD44, as shown in the literature in general, with some authors reporting positivity rates approaching 100% [23], a rate far above that expected for stem cells. In addition, CD44 expression and MYC expression were not associated in neoplastic tissues. Nevertheless, CD44 variants are believed to be better markers for stem cells, in association or not with other markers such as CD133, CD24, CD40, and *α*2*β*1 integrin. While the number of commercially available antibodies against CD44v is still limited, ongoing research is likely to expand the range of options in the near future [50].

Our study was limited by the small sample size, by the use of archived specimens, and by the short time of follow-up. However, our results are supported by other studies showing high rates of CD44 positivity in prostate tissues, contrary to what would be expected for a stem cells marker. Our method of CD44 analysis was similar to that of Tei et al. (2014) [35], as were the results, despite demographic differences between the two samples (Brazil and Japan). Our study does not pretend to be conclusive regarding the role of CD44 in the prognosis of prostatectomized patients, or the potential of CD44 as a stem cell marker. To clarify the issue, studies on much larger samples would be useful or, considering the wide scope of studies conducted so far, a retrograde review of the data and a standardization of the plotting procedures.

## 5. Conclusion

CD44 expression was stronger in inflammatory and nonneoplastic cells than in tumor cells. Within the latter, expression was stronger in well-differentiated than poorly differentiated tumors. The loss of CD44 expression on immunohistochemistry was weakly associated with greater tumor aggressiveness (lower preoperative PSA levels and Gleason scores), but no correlation with biochemical recurrence was observed. The wide range of panCD44 expression in prostate tissues would not detect the minute minority of cancer stem cells by immunohistochemistry.

## Figures and Tables

**Table 1:** Summary of immunohistochemical findings for CD44, plotted according to negative scores (0–3) and positive scores (4–6).

	Inflammatory cells	Nonneoplastic cells	Neoplastic cells
Total	69	68	65
Negative	40 (58.0%)	41 (60.3%)	57 (87.7%)
Positive	29 (42.0%)	27 (39.7%)	8 (12.3%)

**Table 2:** Staining for CD44 in neoplastic cells stratified into negative (0–3) and positive (4–6).

Factor	CD44-negative	CD44-positive	*p* value	OR (95% CI)
*PSA*				
<10	35 (61.4%)^*∗*^	7 (87.5%)	0.242	0.227
≥10	22 (38.6%)	1 (12.5%)		(0.026–1.975)
*Gleason score*				
≤6	31 (54.4%)	6 (75.0%)	0.449	0.397
≥7	26 (45.6%)	2 (25.0%)		(0.074–2.139)
*Tumor stage*				
pT2	42 (73.7%)	7 (87.5%)	0.668	0.400
pT3	15 (26.3%)	1 (2.5%)		(0.045–3.527)
*Surgical margins*				
Free	34 (59.6%)	6 (75.0%)	0.471	0.493
Compromised	23 (40.4%)	2 (25.0%)		(0.091–2.659)
*BR*				
Absent	22 (68.7%)	20 (69.0%)	1.0	0.990
Present	10 (32.3%)	9 (31.0%)		(0.334–2.930)

PSA = prostatic specific antigen; BR = biochemical recurrence; OR (95% CI) = odds ratio and 95% confidence interval; ^*∗*^columns percentage. The level of significance was evaluated with Fisher's exact test.

## Supplementary Materials

Supplementary Materials Supplementary material contains revised articles on CD44 as a prognostic factor in prostatectomies from patients with adenocarcinoma. Table S1: immunohistochemical evaluations of CD44 expression in prostatectomies. Table S2: studies on biochemical recurrence of prostate cancer evaluating CD44 expression in radical prostatectomy.
