# Supplementary material for: PanCD44 Immunohistochemical Evaluation in Prostatectomies from Patients with Adenocarcinoma
Source: Biomed Res Int. 2018 Feb 26;2018:2061268. doi: 10.1155/2018/2061268 (PMC5846379; doi:10.1155/2018/2061268)
Supplement: Supplementary Materials — Supplementary material contains revised articles on CD44 as a prognostic factor in prostatectomies from patients with adenocarcinoma. Table S1: immunohistochemical evaluations of CD44 expression in prostatectomies. Table S2: studies on biochemical recurrence of prostate cancer evaluating CD44 expression in radical prostatectomy. [file 2061268.f1.pdf]

Supplement

Table S1: Immunohistochemical evaluations of CD44 expression in prostatectomies.

Author	N	Clone	Plot	Cut-off	Malignant
Nagabhushan et al., 1996 (43)	50	GKW.A3	0, 1, 2 and 3	> 1	60%
Noordzij et al., 1997 (11)	97	SFF2	<10%, 10-25%, 25-50% and >50%		86%
Kallakury et al., 1998 (47)	106		Intensity: weak, moderate, intense. Extension: focal <10%, regional 11-50%, diffuse>50%	Weak and diffuse; moderate and regional or diffuse; Intense and any extension.	53%
Marzo et al., 1998 (10)	94	2C5<br>H4C4<br>U9M2	0 = 0-5%<br>1 = 6-25%<br>2 = 26-50%<br>3 = 51-75%<br>4 = >75%<br>0 = absent<br>1 = weak<br>2 = moderate<br>3 = strong<br>4 = intense	>6+	80%
Paradis et al., 1998 (22)	38		Focal <20%<br>Diffuse >20%	>20%	37%
Brewster et al., 1999 (29)	76	DF1485	Negative 0%<br>Focal ≤ 10%<br>Regional 11-50%<br>Diffuse >50%	>10%	36%
Aaltomaa et al., 2002 (46)	77	2C5	Continuous	>25%	66%
Lazari et al., 2013 (48)	135	DF1485	1+ = <10%<br>2+ = 10-50%<br>3+ = >50%		3+ = 37.8%
Tei et al., 2014 (35)	160		Extension:<br>0 = 0-5%<br>1 = 6-25%<br>2 = 26-75%<br>3 = 75-100%<br>Intensity:<br>1 = weak<br>2 = intermediate<br>3 = strong	>3	28.2%

Empty cells indicate unavailable data.

Table S2: Studies on biochemical recurrence of prostate cancer evaluating CD44 expression in radical prostatectomy.

Author	N	T	C.O.	BR	2x2	LR	Cox (HR-IC-p)
Noordzij et al., 1997 (11)	29	81	1.0	41%	0.002		0.381 (0.179-0.974) *p*=0.012
Kallakury et al., 1998 (47)	99	39.9	0.4	26%	0.02		NS
Paradis et al.,1998 (22)	38	17.1		26%		NS	
Brewster et al., 1999 (29)	76	38	0.2	30%	0.002[a]	0.0465	NS
Aaltomaa et al., 2002 (46)	77	49.2	0.2	30%	NS		NS
Tei et al., 2014 (35)	160	51	0.2	25%			HR - 2.09 *p*=0.056[b]

N=number of patients submitted to prostatectomy and follow-up; T=mean or median duration of follow-up (months); C.O.=cut-off for biochemical recurrence (PSA in ng/mL); BR=percentage of patients with biochemical recurrence; 2x2=level of significance (*p*-value) in univariate analysis with a 2x2 table using the chi-squared test or Fisher's exact test; LR=Log Rank statistical significance (*p*-value); Cox (HR-IC-p) = Cox regression with Hazard Ratio at the 95%confidence interval and *p*-value; NS=statistically nonsignificant; [a]=data extracted from the text and recalculated; [b]=univariate Cox regression. Empty cells represent values not available or not calculated.
